# The ecological framework of population health – Adding public trust as a forcing factor for U.S. life expectancy and COVID-19 mortality

**DOI:** 10.1016/j.puhip.2026.100791

**Published:** 2026-04-24

**Authors:** Ross Arena, Shuaijie Wang, Colin Woodard, Tanvi Bhatt, Nicolaas P. Pronk

**Affiliations:** a Department of Physical Therapy, University of Illinois Chicago, Chicago, IL, USA; b Healthy Living for Pandemic Event Protection (HL – PIVOT) Network, Chicago, IL, USA; c HealthPartners Institute, Minneapolis, MN, USA; d Nationhood Lab, Pell Center for International Relations and Public Policy, Salve Regina University, Newport, RI, USA; e Department of Health Policy and Management, University of Minnesota, Minneapolis, MN, USA

**Keywords:** Public health, Social media, Health messaging, Lifestyle behaviors, Chronic disease

## Abstract

**Objectives:**

What are the factors that *set the stage* for health status and outcomes in the United States (U.S.)? This complex question is rarely considered in a comprehensive way. The current study employs an artificial intelligence analysis to assess the accuracy of adding a measure of social capital (i.e., public trust) to the Ecological Framework of Population Health in predicting U.S. county-level life expectancy and COVID-19 mortality rates.

**Study design:**

Descriptive, cross-sectional, retrospective analysis.

**Methods:**

The current study utilized several U.S. county-level datasets representing the Ecological Framework of Population Health, including measures of culture, politics, policy, socioeconomics, lifestyle behaviors, and both chronic disease risk factors and diagnoses. A social media generated index of social capital, i.e., public trust, was added to the framework as a crosscutting variable to determine its efficacy in predicting county-level life expectancy and COVID-19 mortality rates using a non-linear artificial intelligence statistical method.

**Results:**

Analysis revealed significance in predicting both life expectancy (R^2^ = 0.803) and COVID-19 deaths (R^2^ = 0.548), with the optimal model employing 27 and 12 features, respectively. The public trust index was retained in both final models, ranking as the 5th and 6th most important predictors for life expectancy and COVID-19 mortality, respectively.

**Conclusions:**

The present study expands the body of work exploring forcing factors of population health by demonstrating the potential utility of a measure of public trust, derived from social media posts and integrated into the Ecological Framework of Population Health, in predicting important U.S. county-level health outcomes.

## 1 Introduction

What are the factors that *set the stage* for health status and outcomes in the United States (U.S.)? This is a complex question and one that is infrequently considered in a comprehensive way in the current public health environment [1]. Rather, in the context of chronic noncommunicable conditions, health surveillance, public health initiatives and healthcare delivery, the focus initiates at the level of poor health behaviors – physical inactivity, poor diet and smoking are the primary unfavorable behaviors [2]. Public health messaging and healthcare interventions intended to change unhealthy lifestyle behaviors then direct attention to downstream factors, following a linear sequence from health risk factors to chronic conditions [3]. Moreover, this reactionary, secondary prevention model manages rather than prevents unhealthy lifestyle behaviors, risk factors, and chronic conditions [2].

To achieve a meaningful and necessary shift toward proactive, primary prevention, a shift that is acknowledged by all stakeholders to be essential to improving population health, we need a better understanding of factors that lie upstream from health behaviors; what makes an individual or group of individuals within a community choose one set of health behaviors over another? The Ecological Framework of Population Health facilitates the exploration of this question [4]. The Framework purports sequential forcing factors upstream from health behaviors, with culture, politics, policy and socioeconomics positioned as core drivers. The Framework also proposes two important throughlines, or crosscutting themes, that interact with every level: power dynamics and social capital. A detailed presentation of the Framework has been published [4], and subsequent artificial intelligence analyses have provided an initial validation of the proposed upstream forcing factors [5].

The Ecological Framework of Population Health is an evolving model, and the assessment of measures to represent key drivers is being considered on an ongoing basis. Using a language-based assessment of generalized trust, Giorgi et al. [6] assessed more than 1.6 billion tweets (collected between 2009 and 2015) to create an index of public trust on a U.S. County level. Public trust is widely recognized as a foundational element and a primary indicator of social capital, which is “represented by networks of relationships among people to live life as a collective in pluralistic societies” [4]. Public trust specifically represents the collective belief that individuals, groups, and institutions will act predictably and fairly, facilitating the cooperation necessary for a well-functioning society [7]. Words associated with trust were correlated with improved health measures collected by the Centers of Disease Control [6]. A valid measure of public trust may add significant predictive value to the Ecological Framework of Population Health developed by Pronk et al. [4] The current study employs an artificial intelligence analysis to assess the accuracy of measures included in the Ecological Framework of Population Health with the addition of a measure of a social media generated public trust index in predicting both life expectancy and COVID-19 mortality rates on a U.S. county-level.

## 2 Methods

The current analysis utilized several U.S. county-level datasets to achieve the study objectives. Table 1 describes source data for independent and dependent measures used in the current study. Source data were linked through zip-code identifiers available in all datasets to create a merged dataset for analysis. The defining characteristics of the four-level American Nations [8] model listed in Table 1 are defined as follows: *Aggressively communitarian* regions have built strong institutions, social services, and regulatory environments paid for by higher taxes on wealth, income, property and business, while the *passively communitarian* ones did so to a lesser degree. The *aggressively individualistic* regions have fewer and weaker institutions, regulatory regimes, and taxes and provide markedly fewer public services. *Passively individualistic* regions are less rigid on these matters but still seek to have a lean public sector. All datasets used in the current analysis are publicly available from the links provided in Table 1 and can be merged through FIPS codes provided in each dataset.

**Table 1:** Source data for independent and dependent variables.

Independent Variable Grouping	Specific Measures	Source
Culture	American Nations Model 4 level grouping: • Aggressively Individualistic (Deep South and Greater Appalachia) • Passively Individualistic (Far West) • Passively Communitarian (Midlands, El Norte) • Aggressively Communitarian (Yankeedom, New Netherland, Left Coast, and First Nation.)	https://www.nationhoodlab.org/the-american-nations-and-the-50-states/https://osf.io/jxsz8/overview?view_only=968233c39a594d14bed3f201b24ec0f8
Trust	Quantifying Generalized Trust in Individuals and Counties Using Computational Linguistic Analysis	https://osf.io/ap8rx/overview
Politics	2022 MRP Ideology Index	https://dataverse.harvard.edu/dataset.xhtml?persistentId=doi:10.7910/DVN/BQKU4M
	Percentage of citizen population aged 18 or older who voted in the 2020 U S. Presidential election.	https://www.countyhealthrankings.org/health-data/methodology-and-sources/data-documentation
Policies	Gross Domestic Product 2019-2022	https://www.bea.gov/data/gdp/gdp-county-metro-and-other-areas
	Percentage of all households that self-responded to the 2020 census	https://www.countyhealthrankings.org/health-data/methodology-and-sources/data-documentation
	2020/2021 Civic Opportunity Index	https://dataverse.harvard.edu/dataset.xhtml?persistentId=doi:10.7910/DVN/TCXRTM
Social, Physical and Economic Environments	Social Vulnerability Index Subtheme Scores 1-4 (2022 Release)	https://www.atsdr.cdc.gov/place-health/php/svi/index.html
Behaviors	2022 Age Adjusted Prevalence of No Leisure-Time Physical Activity, Smoking, Bing Drinking, and Short Sleep Duration (2024 Release)	https://www.cdc.gov/places/
Health Risk Factors	Prevalence of Obesity, Diabetes, Hypertension, High Cholesterol, Depression, Arthritis, Cognitive Disability, Any Disability, Frequent Mental and Physical Distress, and Fair to Poor Health Rating (2024 Release)	https://www.cdc.gov/places/
Social Capital	2022 Social Association Rate: Number of membership associations per 10,000 population	https://www.countyhealthrankings.org/

**Dependent Variable Grouping**	**Specific Measure**	**Source**
Outcomes	Life expectancy average 2020-2022 (2025 release)	https://www.countyhealthrankings.org/health-data
	County-level Cumulative COVID deaths per 100k	https://healthdata.gov/Health/COVID-19-Community-Profile-Report/gqxm-d9w9/about_data

### 2.1 Subject protection

HealthPartners Institute Research Subjects Protection Program determined that this study is exempt from IRB review and ongoing oversight under 45 CFR Part 46 as it involves the analysis of existing, publicly available data sets.

### 2.2 Machine learning

#### 2.2.1 Preprocessing

**Fig. 1:**
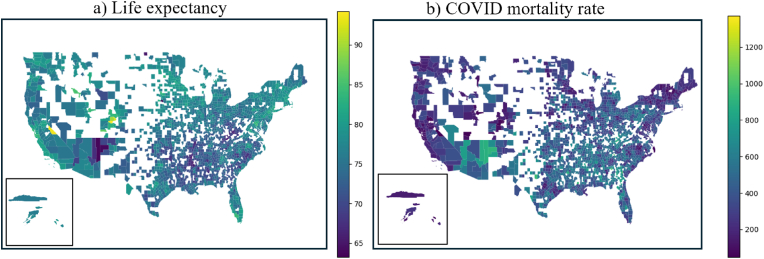
County-level life expectancy and COVID mortality rate (deaths per 100k). **Legend:** COVID, Coronavirus disease.

**Fig. 2:**
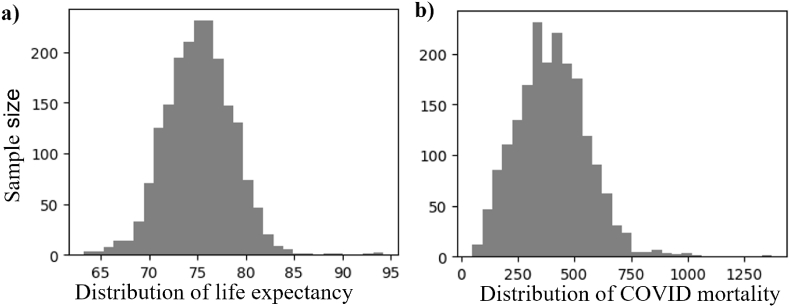
Distribution of life expectancy and COVID mortality rate (deaths per 100k). **Legend:** COVID, Coronavirus disease.

Before model training, several essential preprocessing steps were applied, including handling missing data, removing duplicates, encoding categorical variables, and scaling numerical features. Missing values were detected in both predictors and outcomes. Given the sufficient sample size (n = 1996) relative to the number of features (n = 32), with more than 60 observations per feature, samples containing any missing values in either predictors or outcomes were removed using dropna function in Pandas. Duplicates were detected and removed using drop_duplicates function. After these steps, the final dataset was 1919 for life expectancy and COVID mortality rate prediction. Their county-level distribution and corresponding histogram were presented in Fig. 1, Fig. 2. All predictors were standardized using StandardScaler method to ensure consistent data scaling. Although tree-based models do not require feature scaling, all predictors were standardized to ensure consistency across candidate models evaluated during preliminary analyses.

#### 2.2.2 Model selection

LightGBM was selected as the primary algorithm based on our preliminary evaluations using all 32 features. We compared six widely used machine learning regressors: Support Vector Machine (SVM) regressor, Adaboost regressor, Random Forest regressor, XGBoost regressor, LightGBM regressor, and Extra trees regressor [[9], [10], [11], [12], [13], [14], [15]] on full feature set. Among these models, LightGBM achieved the lowest mean absolute error (MAE) without signs of overfitting. Overfitting was evaluated by comparing performance on the training set and the independent test set. The model showed comparable MAE values between the two datasets, indicating no substantial overfitting. As a gradient boosting framework based on decision tree algorithms, LightGBM is recognized for its high computational efficiency, low memory usage, and robust predictive performance, making it particularly suitable for regression tasks in large-scale datasets.

#### 2.2.3 Model training

The dataset was split into a training set (70%) and a test set (30%). Hyperparameter optimization for the LightGBM Regressor was performed using randomized search with 5-fold cross-validation, tuned parameters include number of trees, maximum depth of trees, learning rate, number of leaves, L1 regularization, subsampling of data instances, and subsample ratio of columns. The last three parameters could help prevent overfitting. Each model was trained using a backward feature selection approach. Starting with all 32 features, we iteratively removed the five features with the lowest importance scores until only seven remained. The importance scores were computed using LightGBM's feature importances attribute. This procedure retained the most predictive variables while progressively reducing dimensionality, improving both generalizability and interpretability.

#### 2.2.4 Model evaluation

To evaluate model performance, MAE, root mean square error (RMSE), and determination coefficient (R^2^), were calculated. Final model performance was evaluated on the held-out test set to assess out-of-sample generalization. The equations for computing MAE, and RMSE are presented below:

$$MAE=\frac{1}{n}\sum_{i=1}^{n}\left|y_{pred,i}-y_{i}\right|$$

$$RMSE=\sqrt{\frac{1}{n}\sum_{i=1}^{n}{\left(y_{pred,i}-y_{i}\right)}^{2}}$$

Where *y*_*pred,i*_ is the predicted value for the *i*-th observation, *y*_*i*_ is the actual value, and *n* is the total number of samples. In this work, MAE is used as the primary metric to evaluate and compare model performance across feature sets (7 to 32 with an interval of 5). The optimal model across different number of features was selected using the feature–MAE curve, and cross-validated using R^2^ and RMSE. A lower MAE indicates better predictive performance, allowing identification of the model that achieved the best balance between accuracy and model complexity.

To assess feature importance, we computed Shapley (SHAP) values for each feature and feature interaction using the training dataset [16] and permutation importances using the test dataset. SHAP analysis quantifies the extent to which the model relies on each predictor when generating predictions. The training set was used rather than the test set to maintain interpretability consistency and accurately reflect the relationships encoded [17] by the model. In contrast, permutation feature importance was estimated using the test set to obtain an unbiased measure of each feature's impact on model generalization. For each feature, we permuted its values while keeping all other features unchanged, resulting in decrease (permutation importance) in predictive performance. Hence, the importance of permutation for each feature can represent the effect of removing that feature's information from the model.

## 3 Results

**Fig. 3:**
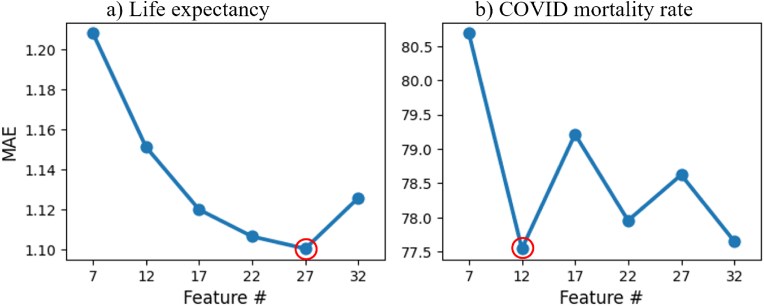
The changes in MAE with different feature counts (#) of life expectancy and COVID mortality rate (deaths per 100k) prediction. The optimal model for each outcome was highlighted by red circle. **Legend:** COVID, Coronavirus disease.

The feature-MAE curve for life expectancy indicated that the performance of LightGBM model improved substantially as the feature count increased from 7 to 27 and then dropped from 27 to 32 (Fig. 3a). Therefore, the 27-feature model with best performance was selected as the optimal model for life expectancy prediction. For the COVID mortality rate, the model performance greatly improved as the feature count increased from 7 to 12 and then fluctuated between 12 and 27 features (Fig. 3b). Hence, the 12-feature model with best performance along with low complexity was selected as the optimal model for COVID mortality prediction. Similar patterns were observed in the R^2^ and RMSE values with different feature counts, which further support the selection of the optimal models (Table 2).

**Table 2:** The R^2^ and RMSE of life expectancy prediction model and COVID mortality rate prediction model with different feature counts (#). The optimal models are indicated in bold.

Feature #	Life expectancy		COVID mortality rate	
	R^2^	RMSE	R^2^	RMSE
32	0.793	1.550	0.549	104.72
27	**0.803**	**1.514**	0.549	104.68
22	0.802	1.517	0.555	103.94
17	0.800	1.527	0.544	105.25
12	0.796	1.539	**0.548**	**104.76**
7	0.766	1.651	0.529	107.01

**Legend:** COVID, Coronavirus disease.

**Fig. 4:**
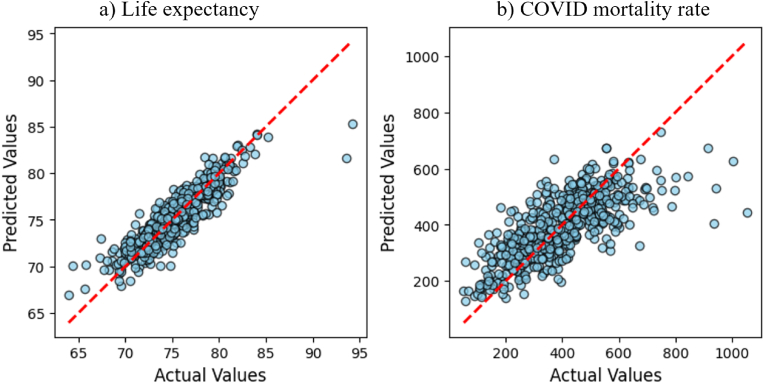
Comparison of actual outcomes and predicted outcomes for a) the optimal life expectancy prediction model (feature count = 27), and b) the optimal COVID mortality rate (deaths per 100k) prediction model (feature count = 12). **Legend:** COVID, Coronavirus disease.

The optimal life expectancy model demonstrated strong predictive capability, explaining 80.3% of the variance (R^2^ = 0.803 in Table 2). The actual and predicted values for individual samples in the test dataset showed strong agreement (Fig. 4a), only two extreme values (>90 yrs) showed greater discrepancy. The optimal COVID mortality model showed moderate performance with R^2^ of 0.548 (Table 2), suggesting this model could only explain ∼55% of the variance in COVID mortality. Greater discrepancy could be also observed between predicted and actual values (Fig. 4b).

**Fig. 5:**
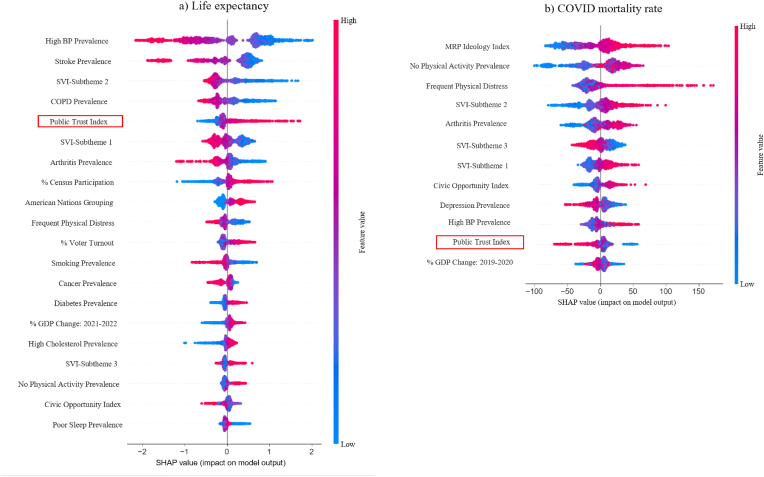
The Shapley (SHAP) values of a) the optimal life expectancy prediction model (top 20 out of 27 features), and b) the optimal COVID mortality prediction model (all 12 features). Positive SHAP values indicate that a contribution toward increasing the predicted outcome (higher life expectancy or COVID mortality rate), while negative SHAP values indicate a contribution toward lower predicted outcome. Larger absolute Shapley values indicate a larger impact on the model output. The features are displayed in decreasing order of mean absolute SHAP value. Horizontal violin plots represent the distribution of SHAP values across all samples in the training set. **Legend:** BP, blood pressure; COPD, chronic obstructive pulmonary disease; COVID, coronavirus disease; GDP, gross domestic product; SVI, social vulnerability index.

**Fig. 6:**
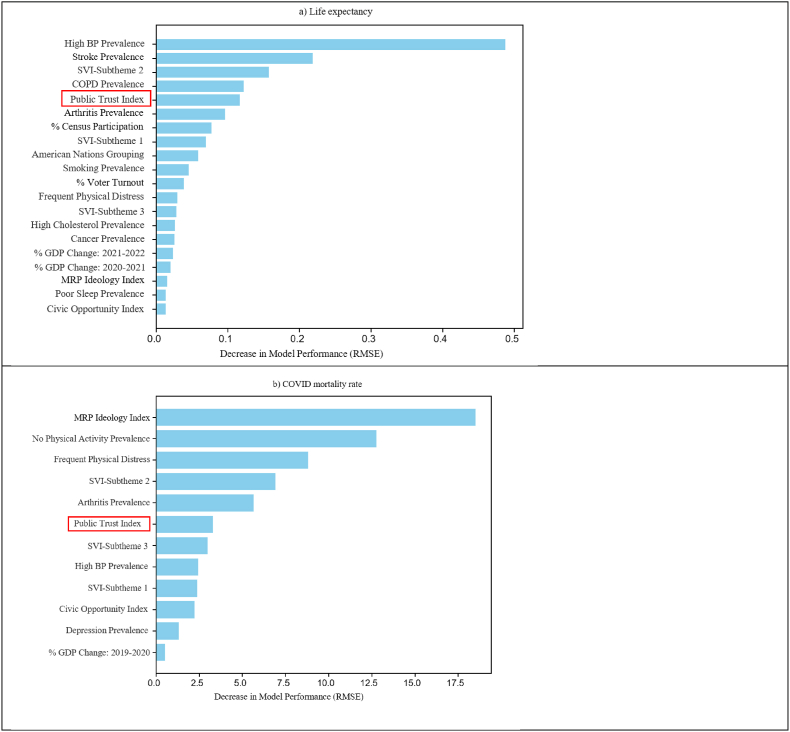
The permutation feature importance of a) the optimal life expectancy prediction model (feature count = 27), and b) the optimal COVID mortality rate prediction model (feature count = 12). Larger permutation values indicate a larger impact on the model output. The features are displayed in decreasing order of permutation value. in the training set. **Legend:** BP, blood pressure; COPD, chronic obstructive pulmonary disease; COVID, coronavirus disease; GDP, gross domestic product; SVI, social vulnerability index.

Feature importance was evaluated using both SHAP values (Fig. 5) and permutation importances (Fig. 6). For both the optimal life expectancy model and COVID mortality model, the top 5 features ranked similarly across these two methods, despite SHAP being computed on the training set and permutation importance on the test set. For the optimal life expectancy model, the top 5 features *are high blood pressure prevalence, stroke prevalence, SVI-Subtheme 2, COPD prevalence,* and the *Public Trust Index*. For the optimal COVID mortality model, the top 5 features are *MRP Ideology Index, no leisure time physical activity prevalence, frequent physical distress prevalence, SVI-Subtheme 2,* and *arthritis prevalence*. Only *SVI-Subtheme 2* appeared in the top five of both life expectancy and COVID mortality models, suggesting that life expectancy and COVID mortality are driven by different features. Notably, *Public Trust Index* was included in both models, its SHAP value ranked 5th in life expectancy model and 6th in the COVID mortality model (Fig. 6), indicating that *Public Trust* played an important role in prediction performance.

**Fig. 7:**
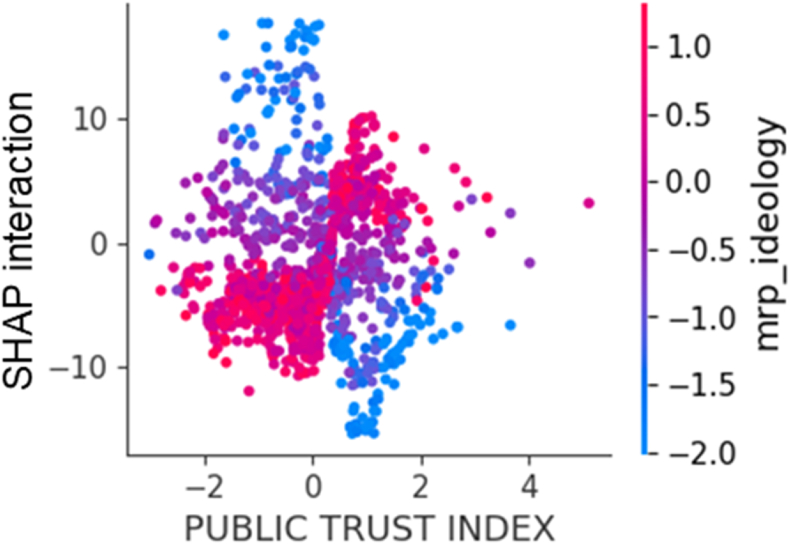
The top Shapley (SHAP) interaction value of the optimal COVID mortality prediction model among interactions between public trust index and other features. **Legend:** COVID, coronavirus disease.

Interaction effects for *Public Trust Index* were estimated using SHAP interaction values. In the optimal life expectancy model, the SHAP interaction values for *Public Trust Index* was close to 0, indicating that interaction terms involving *Public Trust Index* contribute minimally to the life expectancy prediction. For the optimal COVID-19 mortality model, only the interaction between *Public Trust Index* and *MRP Ideology Index* showed a meaningful effect. As shown in Fig. 7, this interaction revealed that when *MRP Ideology Index* was low, higher *Public Trust Index* was associated with lower COVID-19 mortality rate, whereas when *MRP Ideology Index* was high, higher *Public Trust Index* was associated with higher COVID-19 mortality rate.

## 4 Discussion

The entire U.S. health landscape, from population level public health efforts to healthcare delivery on an individual, practitioner to patient interaction level, would benefit from fresh perspectives. Given the disconcerting surveillance data on health behaviors, chronic conditions and life expectancy, a strong case can be made for novel approaches to reverse longstanding trends and future projections based on the current trajectory [[18], [19], [20], [21], [22]]. The Ecological Framework of Population Health [4] assessed in the current investigation provides a needed and novel approach to this complex issue, significantly contributing to this field of study.

In order to develop better approaches to health messaging and delivery, it is necessary to identify significant forcing factors that lie upstream from health behaviors and set the stage for the current and future landscape from population to individual levels. The Ecological Framework of Population Health [4] contributes to this effort by identifying distinct regional U.S. cultures that help explain observed variations in regional politics, policy and socioeconomic status [1,[23], [24], [25]]. These forcing factors are posited to drive downstream lifestyle health behaviors that, when unfavorable, significantly increase the risk of chronic disease, and decreased life expectancy. Two factors, power dynamics and social capital, potentially influence every stage of the framework. The complex interactions amongst levels of the framework and throughlines do not lend themselves to linear predictive assessment, which is why we employed complex artificial intelligence analyses to test the potential predictive ability of the framework [5,26]. By identifying some of the forcing factors that drive U.S. health, we hope to have contributed to a reimagination of health messaging and intervention models. This work is still in its infancy, however, and the Framework requires further exploration and refinement to identify the most useful forcing factors and measurements of their influence.

Giorgi et al. [6] set out to “quantify generalized trust” in individuals and counties across the U.S. To create an index of public trust, language observations of greater than 16,000 Facebook users, in conjunction with a generalized trust score (self-report), were assessed. Their group then used it to analyze more than 1.6 billion geotagged tweets produced between 2009 and 2015, producing estimates of public trust in more than 2000 U S. counties. On a county-level, Giorgi et al. [6] found generalized trust was highest where person-level tweets contained more positive language, such as “love, we, and friends”, and less negative language, such as “hate and stupid.” Mapping the available counties demonstrated clear regional patterns in relative social trust. Using county-level CDC surveillance data, the authors demonstrated higher trust was significantly correlated with higher lifespan and lower self-rated health, obesity and smoking prevalence. The authors concluded that this work provides a model to validly track generalized trust in the U.S. that can be used as a tool for surveillance on a population level.

The current analysis extends the validation and potential value of the measure of generalized trust proposed by Giorgi et al. [6] into the realm of predicting population health outcomes. An initial correlation between the measure of public trust and certain health behaviors and outcomes corroborates the Giorgi and colleagues’ findings [6]. Given the complexity of these relationships, isolated linear regressions and correlations are unable to properly measure how these factors interact and, thus, cannot determine causation. Herein, a complex artificial intelligence method was used to comprehensively assess how well the Ecological Framework of Population Health -- in combination with the proposed generalized measure of public trust -- can predict county-level life expectancy and COVID-19 mortality. These findings indicate that this measure of public trust adds significant predictive value to the Ecological Framework for both assessed outcomes. The results were especially robust in regard to county-level life expectancy where it ranked 5th of 27 predictors in the final model. This life expectancy model, which also included measures from all Ecological Framework Categories, had a predictive accuracy of more than 80%. The COVID-19 mortality model, while significant, had an optimal prediction accuracy of 55%, indicating the presence of additional, unidentified factors. Of note, political ideology and generalized public trust were both prominent predictors for COVID-19 mortality. Lower COVID-19 mortality was clearly linked with higher vaccine uptake [27], which was modulated by political views and trust in the federal government public health messaging [28]. Our findings further support these observed connections between public trust and political ideology. In fact, with respect to COVID-19 mortality, the interaction between the Public Trust and MRP Ideology indices was the only significant observation in the current analysis. This would indicate regional differences in public trust should be further explored in the context of political affiliation and viewpoints. Moreover, physical inactivity has been linked to untoward COVID-19 outcomes [29], so much so that the CDC recognized this unhealthy lifestyle behavior as a significant risk for those infected with COVID-19 [30]. The present study found physical inactivity to be the second most important predicator of COVID-19 mortality, behind political ideology. While the nation has moved past the COVID-19 health crisis, there are still important lessons to be learned as future viral pandemics are likely to occur. The findings from the current study may provide insight on how to address future health crises and optimize outcomes.

Previous work dating back more than a decade highlights the potential value of social media for health surveillance and promotion. In 2014, Young [31] emphasized the potential value and limitations of using social media “big data” to track health behaviors. Young noted that the future use of social media “big data” for health surveillance and intervention purposes requires: 1) interdisciplinary collaborative teams; and 2) large, readily available and regularly updated datasets. Recent publications collectively reviewed available evidence supporting the use of social media posts for health behavior assessment found they continue to show promise as a general concept [32], although challenges in application remain (e.g., estimation biases derived from types of posts and acceptability influencing frequency and content) [33]. In 2022, Hunt and Linos [34] called upon the CDC and other state and local agencies “to continue to optimize and rigorously evaluate the use of social media for health promotion”.

The current analysis assesses the ability of general positivity or negativity of social media, as an indicator of public trust, to predict important U.S. County-level health outcomes. Such an approach aligns well with the Ecological Framework of Population Health by Pronk et al. [4], in which cultural differences and views are the foundation for downstream health behaviors and outcomes. While interest in the utility of social media content for tracking and influencing health behaviors has been of interest for several years, the "big data” surveillance regime proposed by Young [31] has yet to be realized. Moving forward, efforts are needed to standardize and validate social media metrics that meaningfully capture significant predictors of population level health behaviors. Once developed, social media metrics should be measured on an annual basis at a refined level in the U.S. (e.g., county-level) and synergized with other health surveillance data. Interdisciplinary teams can then use comprehensive data sets to further improve health surveillance accuracy and conceive of more effective public health messaging campaigns that are customized for the unique regional/community phenotypes.

Beyond the use of social media content for population level health surveillance, the opportunity to deliver health interventions through this platform presents a significant opportunity. Evidence indicates that social media health interventions hold promise [35,36], although more research is needed to determine optimal models of delivery and messaging. The public trust index assessed in the current study provides an example of work needed to determine optimization of social media health interventions, as effectiveness of messaging will likely vary depending on regional cultures, belief systems and viewpoints. Exploration of social media messaging that resonates on a regional/community level is needed – a one-size-fits-all approach to social media health interventions is unlikely to be successful. Given the findings of the current analysis, exploring how to develop health messaging and interventions that would resonate with U.S. regions that have higher social media negativity, and lower public trust would be particularly important.

There are inherent limitations to the current study. First, only 1919 counties had sufficient public trust data to include in the analysis. Fig. 1 illustrates where gaps in data availability are most prominent. While our findings are compelling as to the potential value of gauging public trust through social media, future work is needed with a more complete county dataset to confirm the findings presented herein. Moreover, the public trust index was derived from social media data collected between 2009 and 2015, providing a historical perspective on U.S. regions assessed as compared to other independent and dependent measures used in the current study, where county-level measures were collected between 2019 and 2023. However, the public trust index likely emulates a behavior pattern similar to the American Nations cultural framework [8]. The underlying values, norms, etc. of dominant regional cultures do not fundamentally change year to year, decade to decade, and rarely century to century.

In this context, the underlying values that are being hypothesized to help explain regional differences, in this case public trust, do not considerably change across regions in a decadal time frame. However, to further understand the importance of measuring public trust in the context of population health, an updated calculation is needed. To this point, Young [31] in 2014 proposed population level data on social media patterns be readily available and regularly updated, akin to other surveillance datasets currently collected by other health-focused federal agencies.

The present study expands the exploration of upstream lifestyle behavior factors by demonstrating the potential utility of public trust as a measure of social capital, derived from social media posts and integrated into the Ecological Framework of Population Health [4], in predicting important health outcomes on a U.S. County-level, in this case life expectancy and COVID-19 mortality. Future analyses with expanded and updated datasets are needed to further refine the model to optimize its use in population health surveillance and messaging.

## Author statements

All authors had access to the data. RA prepared the initial draft of the manuscript. RA and SW prepared data analysis. All authors provided critical revisions and new content to the manuscript draft.

## Ethical statement

HealthPartners Institute Research Subjects Protection Program determined that this study is exempt from IRB review and ongoing oversight under 45 CFR Part 46 as it involves the analysis of existing, publicly available data.

## Funding source

None.

## Declaration of competing interest

The authors declare that they have no known competing financial interests or personal relationships that could have appeared to influence the work reported in this paper.
